# Supplementary material for: Adult-onset deletion of ATP13A2 in mice induces progressive nigrostriatal pathway dopaminergic degeneration and lysosomal abnormalities
Source: NPJ Parkinsons Dis. 2024 Jul 20;10:133. doi: 10.1038/s41531-024-00748-5 (PMC11271504; doi:10.1038/s41531-024-00748-5)

Supplementary Materials – Erb et al.

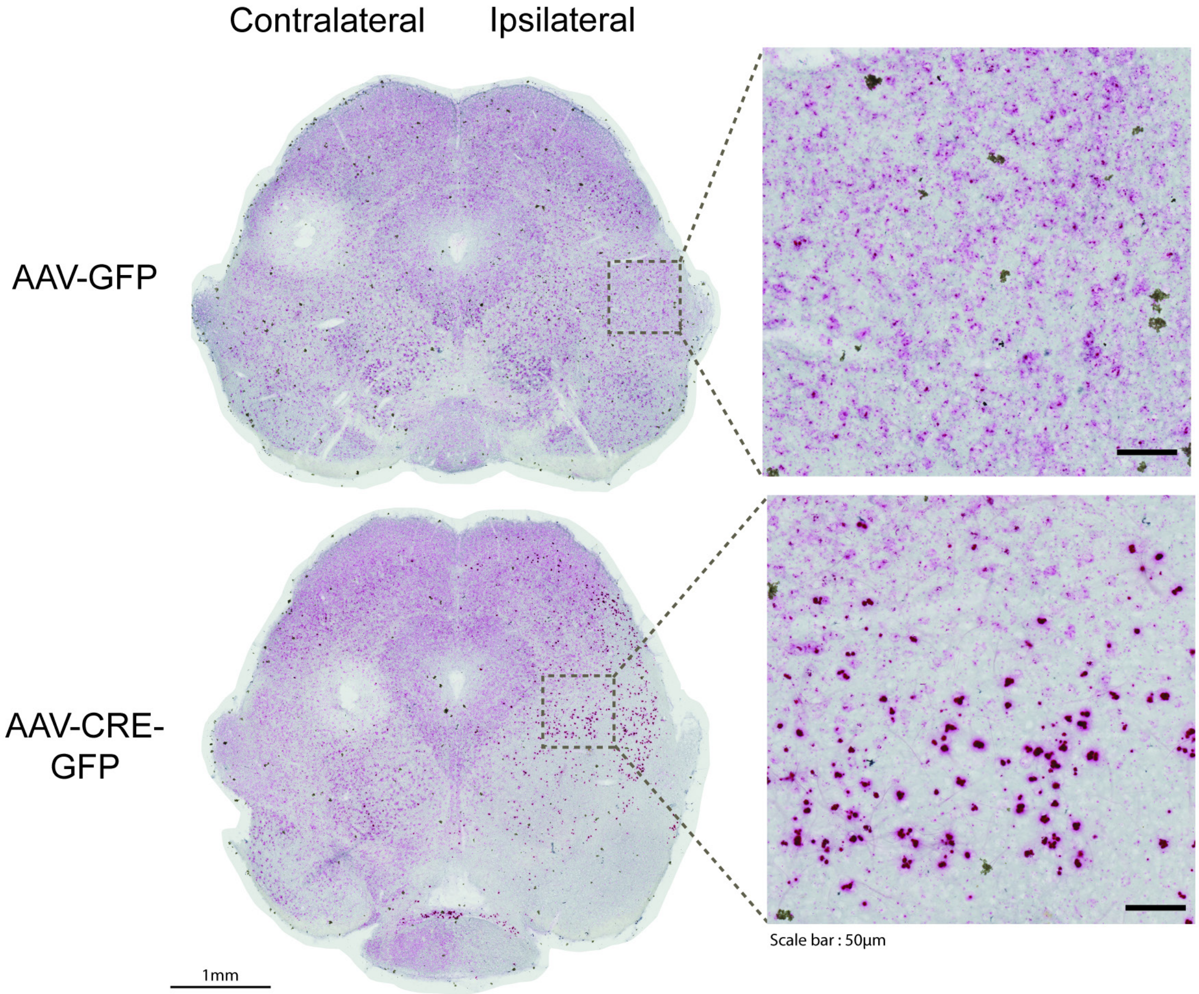

Supplementary Figure 1. *ATP13A2* KO in the ventral midbrain induces upregulation of ATP13A2 mRNA transcript in cells at the periphery. Representative images of BaseScope™ *in situ* hybridization signal recognizing exons 2-3 of ATP13A2 mRNA transcript in ipsilateral and contralateral midbrain tissue sections of *ATP13A2* floxed KO mice injected with AAV-GFP or AAV-Cre-GFP at 3 months. Scale bar: 1 mm. High magnification images indicate individual cells from the ipsilateral midbrain. Scale bar: 50 μm. Notice the selective removal of ATP13A2 signal in the ipsilateral SN of AAV-Cre-GFP mice, yet a robust increase in signal in some cells at the periphery of the ventral midbrain of these mice.

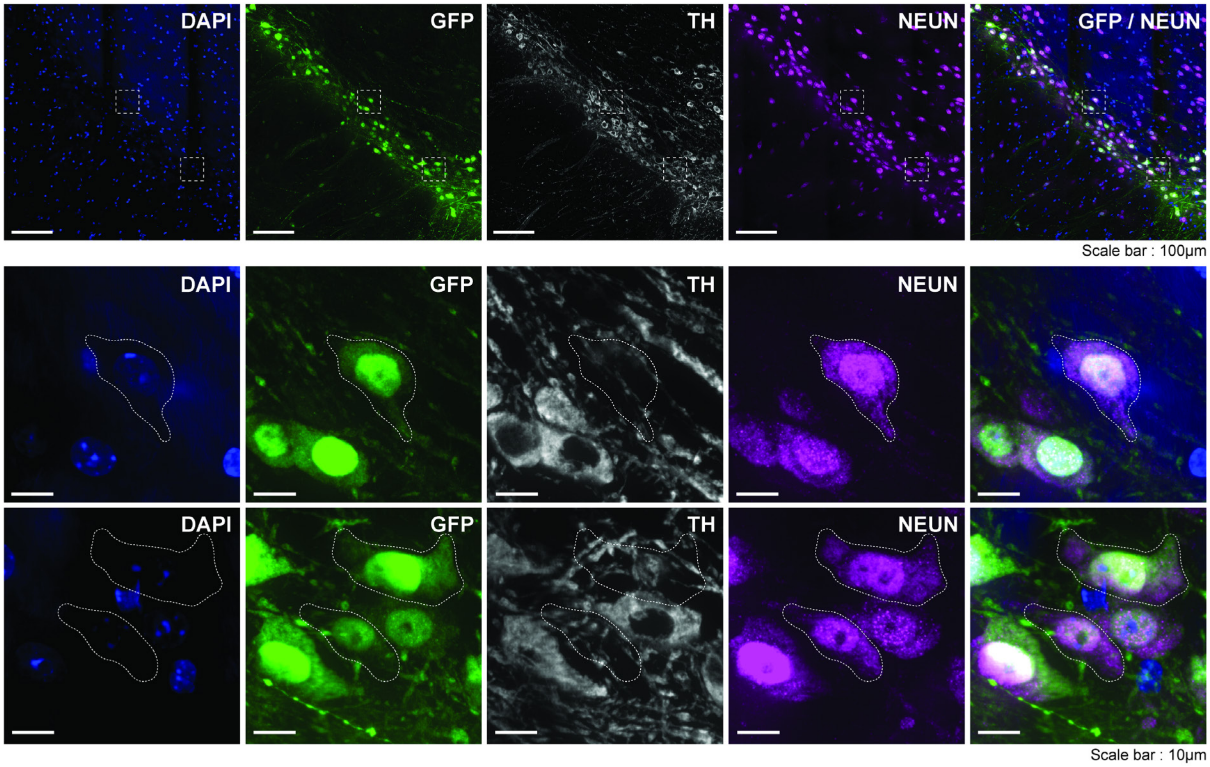

Supplementary Figure 2: Cre-GFP is selectively expressed in NeuN-positive neurons in the substantia nigra. Representative images of NeuN, TH and GFP immunostaining in the ipsilateral SN of AAV-Cre-GFP injected mice at low (upper panels) or high magnification (lower panels). Cre-GFP colocalizes in many TH+ neurons and the majority of NeuN+ neurons. Scale bars: 100 µm or 10 µm.

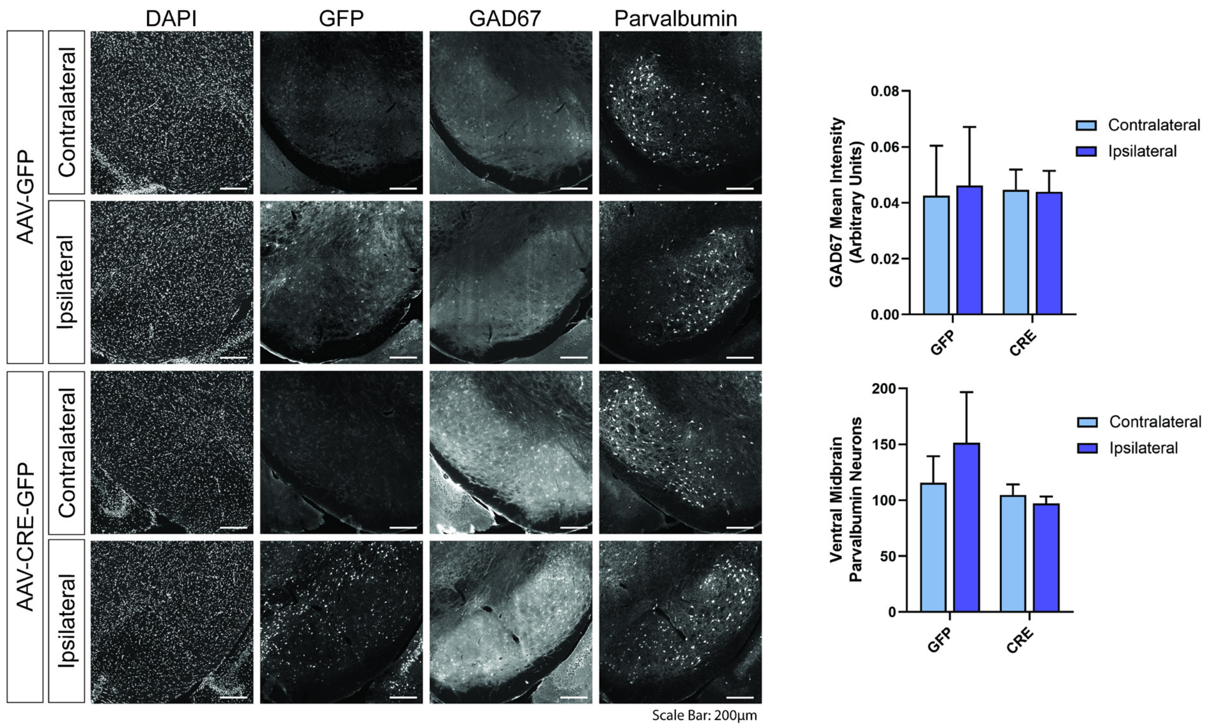

Supplementary Figure 3. Loss of *ATP13A2* does not induce degeneration of GAD67-positive or parvalbumin-positive non-dopaminergic neurons in the substantia nigra. Confocal immunofluorescent images of GAD67, parvalbumin and GFP in the ipsilateral and contralateral SN from *ATP13A2* floxed KO mice injected with AAV-Cre-GFP or AAV-GFP vectors after 10 months. Scale bars: 200 µm. GAD67-positive neuropil fluorescence intensity or parvalbumin-positive neuron number in the SN were analyzed using Cell Profiler image analysis software. Bars represent mean ± SEM, *n* = 4 mice per group. No significant differences between groups are detected by one-way ANOVA.

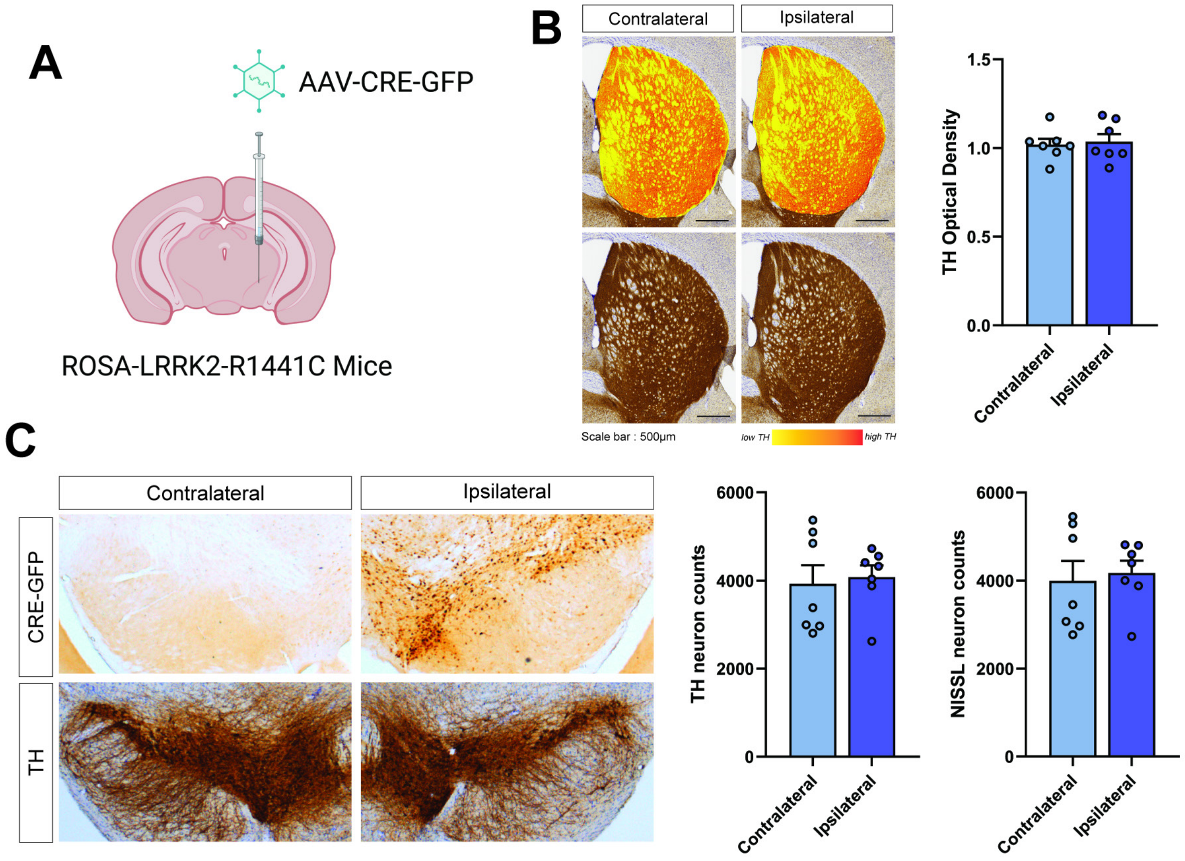

Supplementary Figure 4. Prolonged Cre-GFP expression in the substantia nigra does not independently induce dopaminergic neurodegeneration in mice over 12 months. (**A**) Homozygous floxed ROSA26-LRRK2-R1441C mice were unilaterally injected with AAV-Cre-GFP vector into the SN and analyzed after 12 months. (**B**) Immunolabeled images of TH-positive nerve terminals in the striatum, with optical density measured in the ipsilateral versus contralateral striatum using HALO analysis software. Scale bars: 500 µm. Bars represent mean ± SEM, *n* = 7 mice per group. (**C**) Immunolabeled images of TH-positive dopaminergic neurons or Cre-GFP-positive cells (labeled with a GFP antibody) in the SN, with neurons counted by unbiased stereological analysis of TH-positive and total Nissl-positive neurons in ipsilateral versus contralateral substantia nigra. Bars represent mean ± SEM, *n* = 7 mice per group. No significant differences between groups are detected by unpaired, Student's *t*-test.

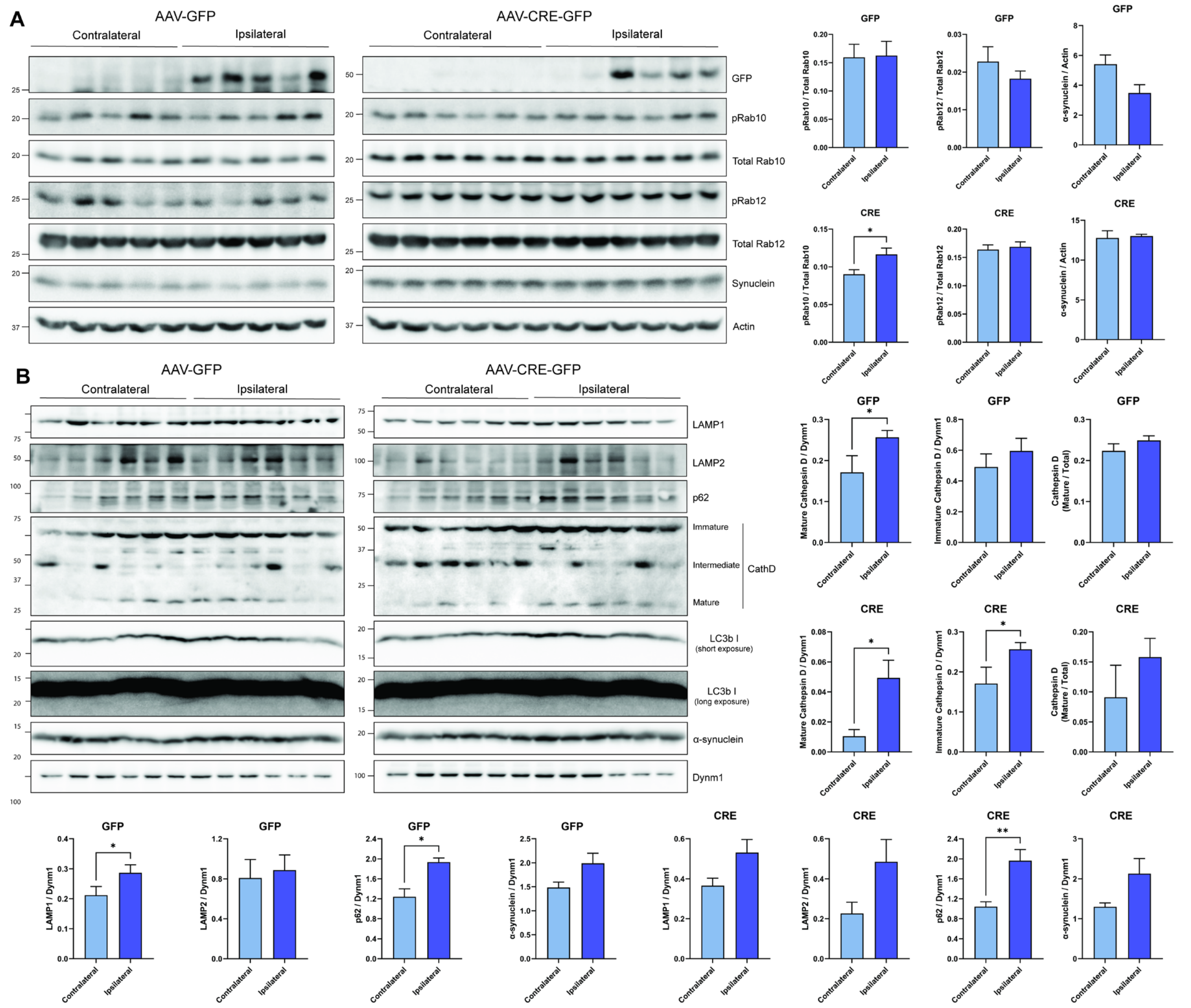

Supplementary Figure 5. Western blot analysis of autophagy-lysosomal pathway proteins in ventral midbrain tissue of *ATP13A2* KO mice at 3 months. (**A**) 1% Triton-X100-soluble ventral midbrain extracts were prepared from *ATP13A2* floxed KO mice injected with AAV-GFP or AAV-Cre-GFP at 3 months. Western blots were probed for autophagy-lysosomal proteins, including pT73-Rab10, total Rab10, pS106-Rab12, total Rab12, total α-synuclein and actin as a loading control. Protein intensities were measured using ImageJ analysis software comparing ipsilateral and contralateral hemispheres for GFP or Cre-GFP mice. Bars represent mean ± SEM, *n* = 5-6 mice per group. *P<0.05 by unpaired, Student's *t*-test. (**B**) 1% Triton-X100-insoluble (RIPA-soluble) ventral midbrain from the same mice. Western blots were probed for autophagy-lysosomal proteins, including LAMP1, LAMP2, p62, cathepsin D species, LC3B I and II,

total α-synuclein and dynamin-1 as a loading control. Protein intensities were measured using ImageJ analysis software comparing ipsilateral and contralateral hemispheres for GFP or Cre-GFP mice. Bars represent mean ± SEM, *n* = 6 mice per group. $^{*}P<0.05$ or $^{**}P<0.01$ by unpaired, Student's *t*-test.

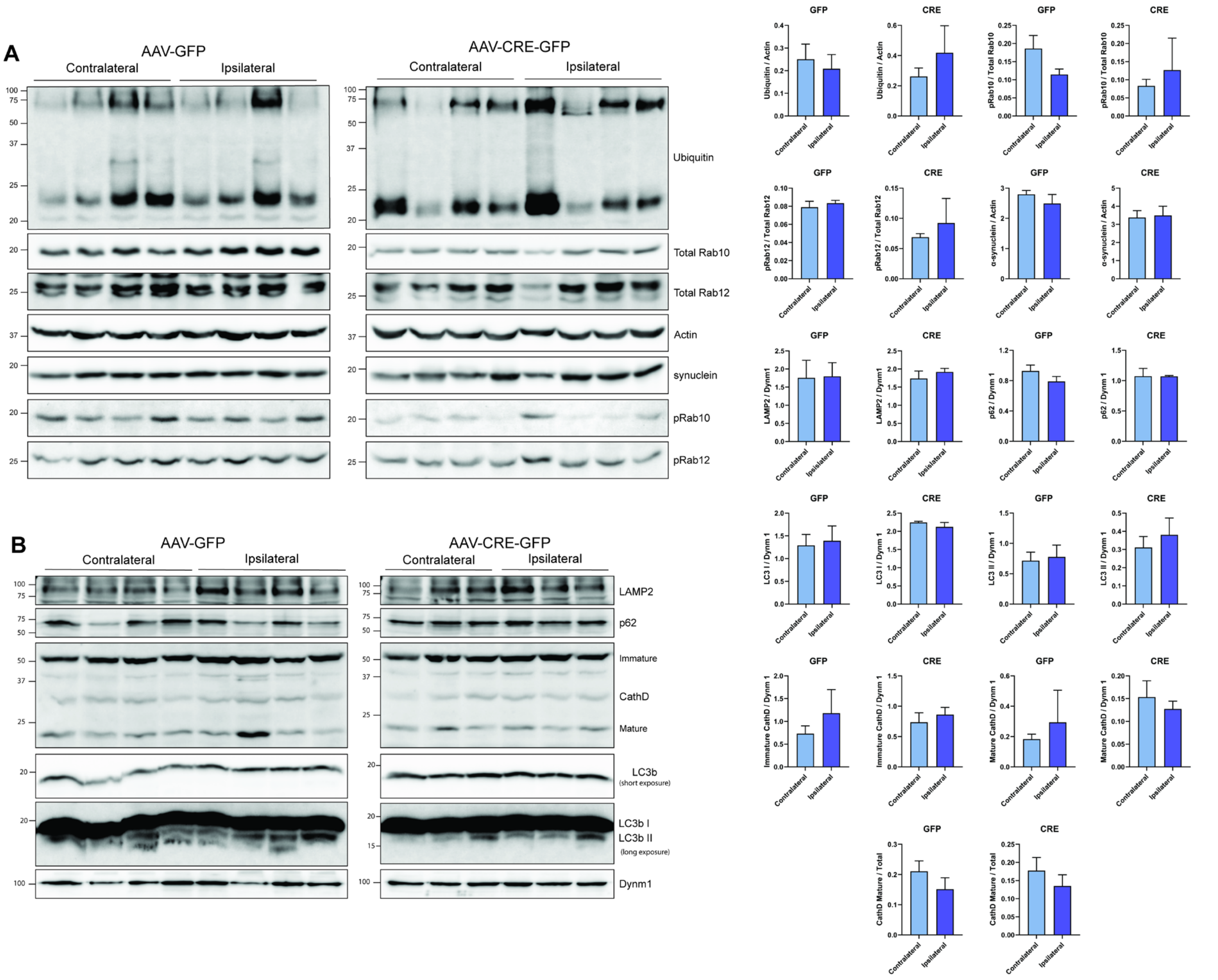

Supplementary Figure 6. Western blot analysis of autophagy-lysosomal pathway proteins in ventral midbrain tissue of *ATP13A2* KO mice at 6 months. (**A**) 1% Triton-X100-soluble ventral midbrain extracts were prepared from *ATP13A2* floxed KO mice injected with AAV-GFP or AAV-Cre-GFP at 6 months. Western blots were probed for autophagy-lysosomal proteins, including total ubiquitin, pT73-Rab10, total Rab10, pS106-Rab12, total Rab12, total α-synuclein and actin as a loading control. Protein intensities were measured using ImageJ analysis software comparing ipsilateral and contralateral hemispheres for GFP or Cre-GFP mice. Bars represent mean ± SEM, *n* = 4 mice per

group. (**B**) 1% Triton-X100-insoluble (RIPA-soluble) ventral midbrain from the same mice. Western blots were probed for autophagy-lysosomal proteins, including LAMP2, p62, cathepsin D species, LC3B I and II, and dynamin-1 as a loading control. Protein intensities were measured using ImageJ analysis software comparing ipsilateral and contralateral hemispheres for GFP or Cre-GFP mice. Bars represent mean ± SEM, n = 3-4 mice per group. No significance by unpaired, Student's *t*-test.

Supplementary Table 1

Figure	Microscope	Objective	Resolution	Acquisition details	Image analysis	Software
1C	ImageXpress Confocal HT Microscope	20X	0.3411 µm/pixel	Z stacks Max Int Proj	qualitative image analysis	N/A
1E	Zeiss Axioscan 7 Microscope Slide Scanner	20X	0.173 µm/pixel	single image	Indica labs Inc. HALO analysis software (Area quantification module)	RRID:SCR_018350 ; https://www.indicalab.com/halo/
1F	Nikon Eclipse Ni-U Upright Microscope	40X	0.075 µm/pixel	single image	qualitative image analysis	N/A
1G	ImageXpress Confocal HT Microscope	60X	0.1125 µm/pixel	Z stacks Max Int Proj	NIH ImageJ (FIJI) (v2.14.0)	RRID:SCR_003070 ; https://imagej.net/
2A	Aperio ScanScope XT slide scanner	20X	0.5 µm/pixel	single image	Indica labs Inc. HALO analysis software (Area quantification module)	RRID:SCR_018350 ; https://www.indicalab.com/halo/
2B	Zeiss Axio Imager.M2	2.5X	2.58 µm/pixel	single image	Micro Bright Field Biosciences StereoInvestigator (stereological cell counts)	RRID:SCR_018948 ; https://www.mbfbioscience.com/products/stereo-investigator
2C	Aperio ScanScope XT slide scanner	20X	0.5 µm/pixel	single image	Indica labs Inc. HALO analysis software (Area quantification module)	RRID:SCR_018350 ; https://www.indicalab.com/halo/
2D	Zeiss Axio Imager.M2	2.5X	2.58 µm/pixel	single image	Micro Bright Field Biosciences StereoInvestigator (stereological cell counts)	RRID:SCR_018948 ; https://www.mbfbioscience.com/products/stereo-investigator
3A	Aperio ScanScope XT slide scanner	20X	0.5 µm/pixel	single image	Indica labs Inc. HALO analysis software (Area quantification module)	RRID:SCR_018350 ; https://www.indicalab.com/halo/

3B	Aperio ScanScope XT slide scanner	20X	0.5 µm/pixel	single image	Indica labs Inc. HALO analysis software (Area quantification module and microglia activation module)	RRID:SCR_018350 ; https://www.indicalab.com/halo/
3C	Aperio ScanScope XT slide scanner	20X	0.5 µm/pixel	single image	Indica labs Inc. HALO analysis software (Area quantification module)	RRID:SCR_018350 ; https://www.indicalab.com/halo/
3D	Aperio ScanScope XT slide scanner	20X	0.5 µm/pixel	single image	Indica labs Inc. HALO analysis software (Area quantification module and microglia activation module)	RRID:SCR_018350 ; https://www.indicalab.com/halo/
4A	Zeiss Axio Imager.M2	20X	0.323 µm/pixel	single image	qualitative image analysis	N/A
4B	Zeiss Axio Imager.M2	2.5X	2.58 µm/pixel	single image	qualitative image analysis	N/A
4C	Zeiss Axio Imager.M2	2.5X	2.58 µm/pixel	single image	qualitative image analysis	N/A
4D	Aperio ScanScope XT slide scanner	20X	0.5 µm/pixel	single image	Indica labs Inc. HALO analysis software (Area quantification module)	RRID:SCR_018350 ; https://www.indicalab.com/halo/
5A	Nikon A1plus-RSi Laser-Scanning Confocal	100X	0.205 µm/pixel	Z stacks Max Int Proj	NIS-Elements analysis software (Nikon)	RRID:SCR_002776; https://www.microscope.healthcare.nikon.com/products/software/nis-elements
5B	Nikon A1plus-RSi Laser-Scanning Confocal	100X	0.205 µm/pixel	Z stacks Max Int Proj	NIH ImageJ (FIJI) (v2.14.0)	RRID:SCR_003070 ; https://imagej.net/
6A	Nikon A1plus-RSi Laser-Scanning Confocal	10X	0.610 µm/pixel	Z stacks (Max Int Proj)	Cell Profiler (v4.2.5)	RRID:SCR_007358 ; https://cellprofiler.org/
6B	ImageXpress Confocal HT Microscope	20X	0.3411 µm/pixel	Z stacks Max Int Proj	qualitative image analysis	N/A

S1	Zeiss Axioscan 7 Microscope Slide Scanner	20X	0.173 µm/pixel	single image	qualitative image analysis	N/A
S2	ImageXpress Confocal HT Microscope	60X	0.1125 µm/pixel	Z stacks Max Int Proj	Cell Profiler (v4.2.5)	RRID:SCR_007358 ; https://cellprofiler.org/
S3 - B	Aperio ScanScope XT slide scanner	20X	0.5 µm/pixel	single image	Indica labs Inc. HALO analysis software (Area quantification module)	RRID:SCR_018350 ; https://www.indicalab.com/halo/
S3 - C	Zeiss Axio Imager.M2	2.5X	2.58 µm/pixel	single image	Micro Bright Field Biosciences StereoInvestigator (stereological cell counts)	RRID:SCR_018948 ; https://www.mbfbioscience.com/products/stereo-investigator

Supplementary Table 2

Immunofluorescence - Primary Antibodies		
**Target**	**Dilution**	**RRID**
TH	1:2000	Novus Biological Cat# N300-109
TH	1:1000	*(Abcam Cat# ab76442, RRID:AB_1524535)*
LAMP2	1:1000	*(Abcam Cat# ab13524, RRID:AB_2134736)*
p62	1:500	*(Progen Cat# GP62-C, RRID:AB_2687531)*
GFP	1:500	*(Thermo Fisher Scientific Cat# A-11122 (also A11122), RRID:AB_221569)*
GFP	1:1000	*(Roche Cat# 11814460001, RRID:AB_390913)*
GFP	1:2000	*(Aves Labs Cat# GFP-1010, RRID:AB_2307313)*
GAD67	1:500	*(Millipore Cat# MAB5406, RRID:AB_2278725)*
parvalbumin	1:500	*(Abcam Cat# ab11427, RRID:AB_298032)*
TFE3	1:500	*(Abcam Cat# ab93808, RRID:AB_10563130)*
Iba1	1:1000	*(FUJIFILM Wako Shibayagi Cat# 019-19741, RRID:AB_839504)*
GFAP	1:1000	*(Sigma-Aldrich Cat# G3893, RRID:AB_477010)*
**Immunofluorescence - Secondary Antibodies**		
**Target**	**Dilution**	**RRID**
goat-anti rabbit AlexaFluor-488	1:500	*(Thermo Fisher Scientific Cat# A-11008 (also A11008), RRID:AB_143165)*
goat-anti rabbit AlexaFluor-546	1:500	*(Thermo Fisher Scientific Cat# A-11010 (also A11010), RRID:AB_2534077)*
goat-anti rabbit AlexaFluor-647	1:500	*(Thermo Fisher Scientific Cat# A-21245 (also A21245), RRID:AB_2535813)*

goat-anti mouse AlexaFluor-488	1:500	*(Thermo Fisher Scientific Cat# A-11029, RRID:AB_2534088)*
goat-anti mouse AlexaFluor-546	1:500	*(Thermo Fisher Scientific Cat# A-11003 (also A11003), RRID:AB_2534071)*
goat-anti rat AlexaFluor-647	1:500	*(Thermo Fisher Scientific Cat# A-21247, RRID:AB_141778)*
goat-anti chicken AlexaFluor-488	1:500	*(Thermo Fisher Scientific Cat# A-11039, RRID:AB_2534096)*
goat-anti chicken AlexaFluor-647	1:500	*(Thermo Fisher Scientific Cat# A-21449, RRID:AB_2535866)*
**Immunohistochemistry - Primary Antibodies**		
**Target**	**Dilution**	**RRID**
TH	1:2000	Novus Biological Cat# N300-109
Iba1	1:1000	*(FUJIFILM Wako Shibayagi Cat# 019-19741, RRID:AB_839504)*
GFAP	1:1000	*(Sigma-Aldrich Cat# G3893, RRID:AB_477010)*
pS129-α-synuclein	1:1000	*(Abcam Cat# ab51253, RRID:AB_869973)*
pSer202/pThr205-Tau (AT8)	1:1000	*(Thermo Fisher Scientific Cat# MN1020, RRID:AB_223647)*
GFP	1:1000	*(Thermo Fisher Scientific Cat# A-11122 (also A11122), RRID:AB_221569)*
α-synuclein	1:1000	*(BD Biosciences Cat# 610787, RRID:AB_398108)*
**Immunohistochemistry - Secondary Antibodies**		
**Target**	**Dilution**	**RRID**
biotinylated goat-anti rabbit	1:1000	*(Vector Laboratories Cat# BA-1000 (also BA-1000-1.5), RRID:AB_2313606)*
biotinylated goat-anti mouse	1:1000	*(Vector Laboratories Cat# BP-9200, RRID:AB_2827937)*
**Western blot - Primary Antibodies**		
**Target**	**Dilution**	**RRID**
GFP	1:500	*(Roche Cat# 11814460001, RRID:AB_390913)*

pThr73-Rab10	1:500	*(Abcam Cat# ab230261, RRID:AB_2811274)*
Rab10	1:500	*(Cell Signaling Technology Cat# 8127 (also 8127S, 8127P, 8127T), RRID:AB_10828219)*
pSer106-Rab12	1:500	*(Abcam Cat# ab256487, RRID:AB_2884880)*
Rab12	1:500	*(Proteintech Cat# 18843-1-AP, RRID:AB_10603469)*
α-synuclein	1:500	*(BD Biosciences Cat# 610787, RRID:AB_398108)*
Actin	1:500	*(Millipore Cat# MAB1501 (also MAB1501R, MAB150X), RRID:AB_2223041)*
LAMP1	1:500	*(Abcam Cat# ab24170, RRID:AB_775978)*
LAMP2	1:500	*(Abcam Cat# ab13524, RRID:AB_2134736)*
p62	1:500	*(Progen Cat# GP62-C, RRID:AB_2687531)*
cathepsin D	1:500	*(Santa Cruz Biotechnology Cat# sc-6487-R, RRID:AB_1120041)*
LC3b	1:500	*(Cell Signaling Technology Cat# 3868 (also 3868S, 3868P), RRID:AB_2137707)*
ubiquitin	1:500	*(Cell Signaling Technology Cat# 3936, RRID:AB_331292)*
Dynamin-1	1:500	*(Thermo Fisher Scientific Cat# PA1-660, RRID:AB_325845)*
**Western blot - Secondary Antibodies**		
**Target**	**Dilution**	**RRID**
goat anti rat	1:5000	*Jackson Immunoresearch Cat# 112-035-175, RRID:AB_2338140*
goat anti mouse	1:5000	*Jackson Immunoresearch Cat# 115-035-174, RRID:AB_2338512*
mouse anti rabbit	1:5000	*Jackson Immunoresearch Cat# 211-032-171, RRID:AB_2339149*
goat anti guinea pig	1:5000	*Abcam Cat# ab97155, RRID:AB_10680107*

Uncropped gel images:

Figure 1D

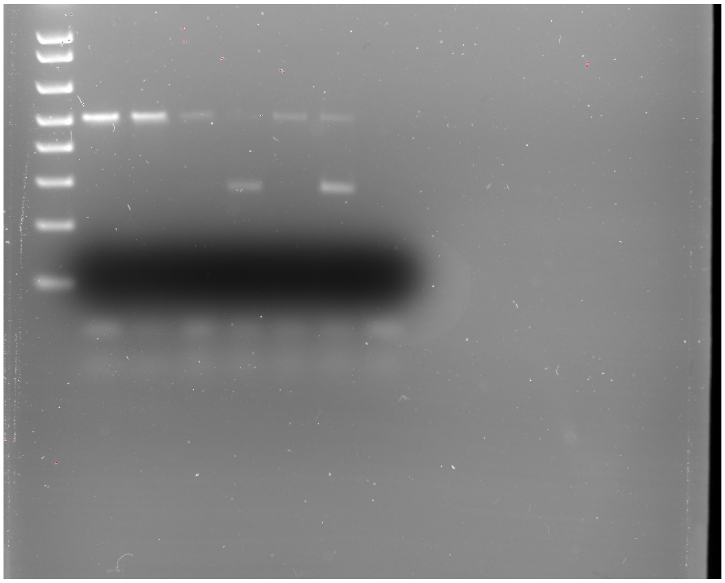

Supplementary Figure 5A

Total α-synuclein

GFP

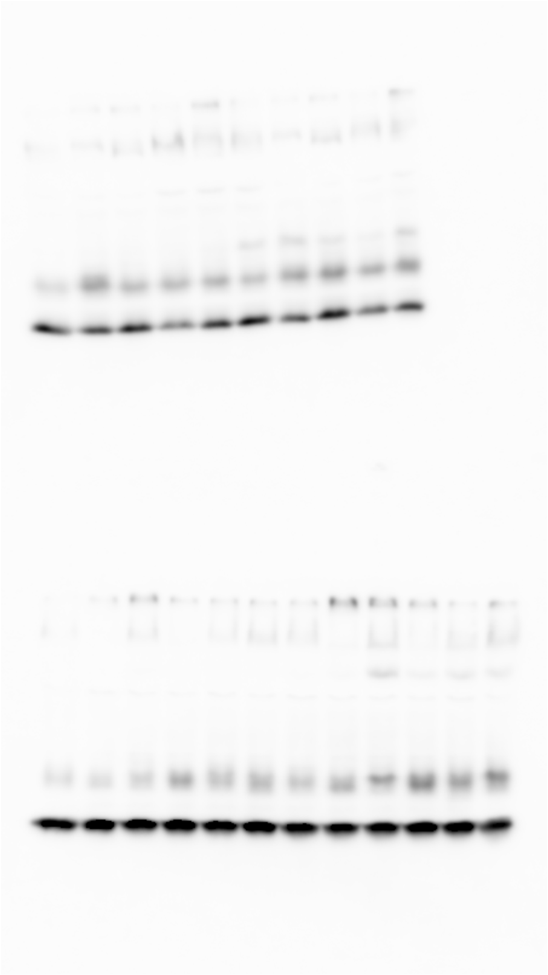

pRab10

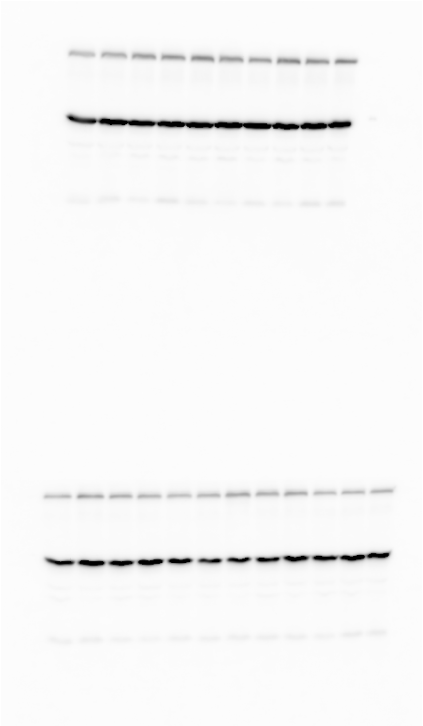

pRab12

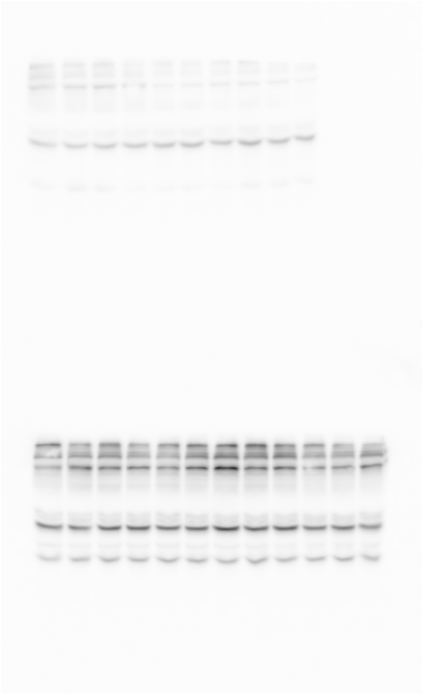

Total Rab10

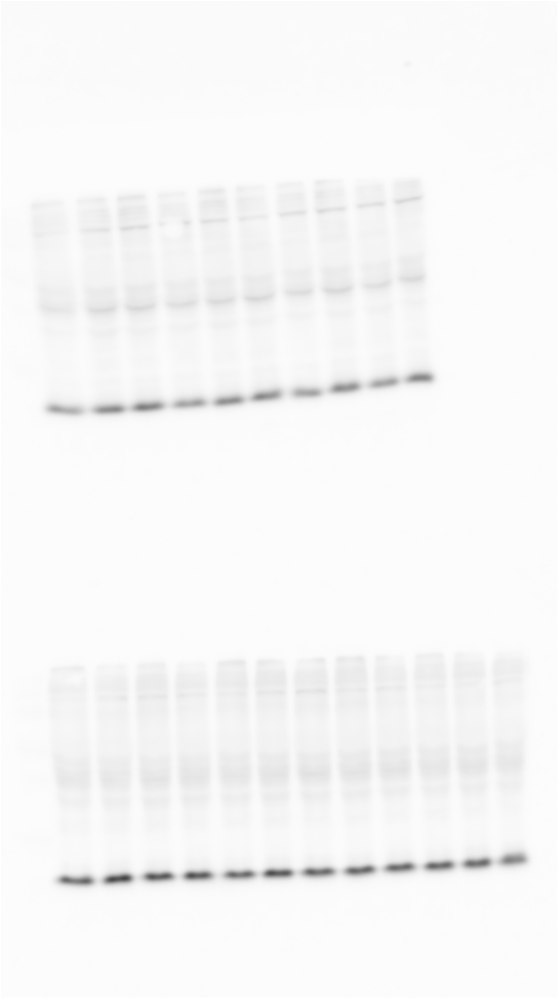

Total Rab12

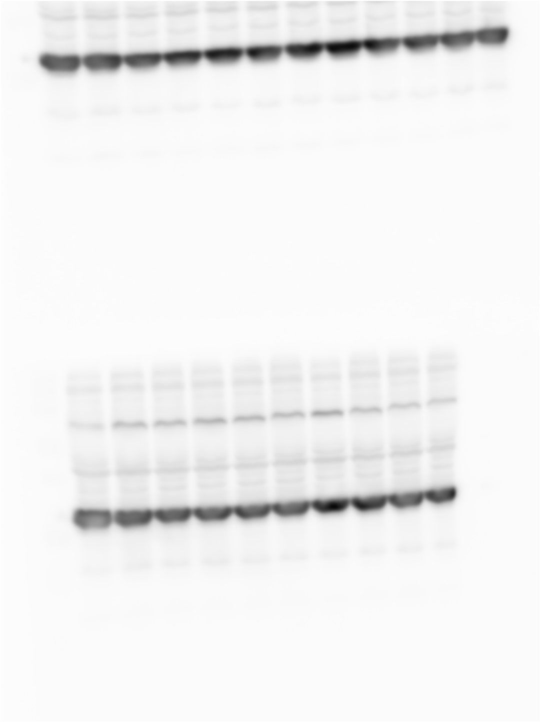

Actin

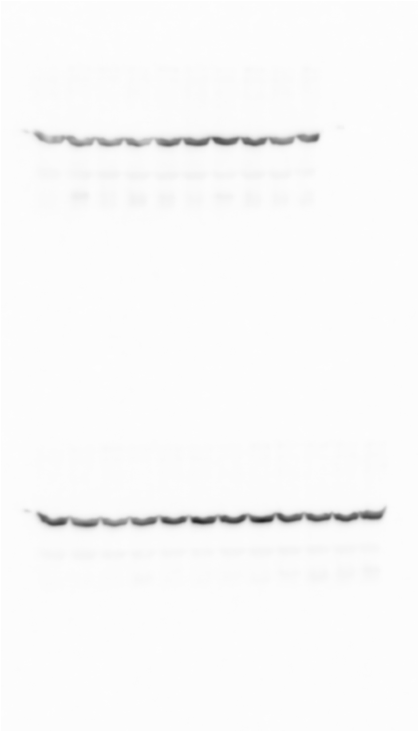

Supplementary Figure 5B

Cathepsin D

LAMP1

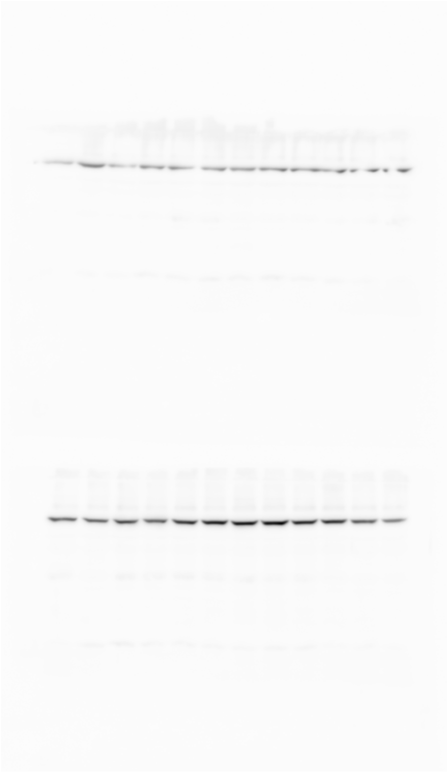

LC3B

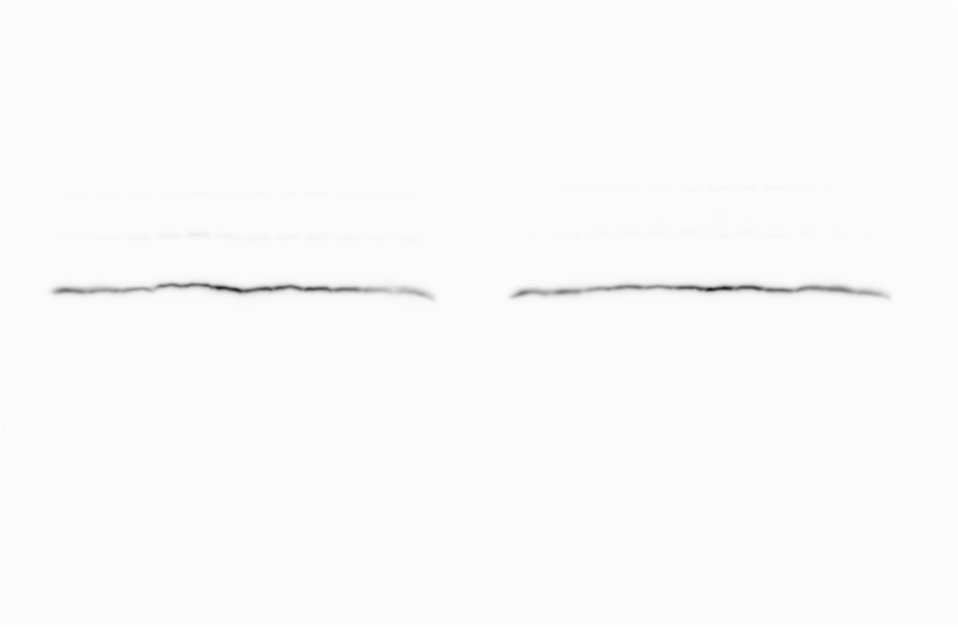

p62

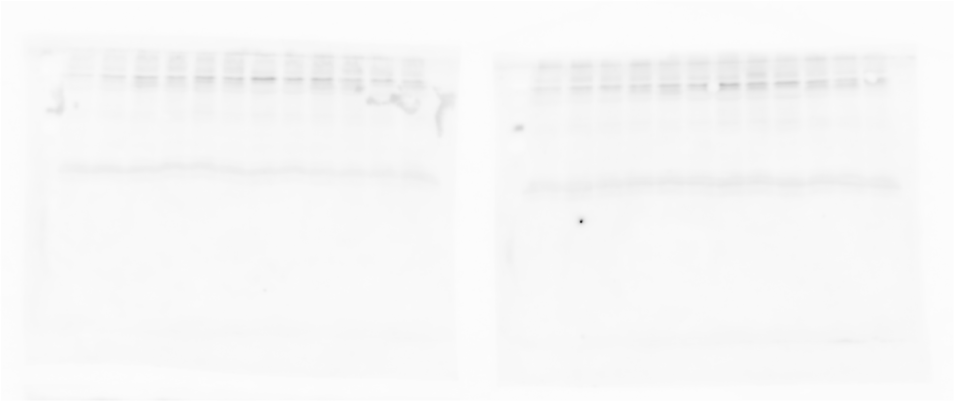

LAMP2

Total α-synuclein

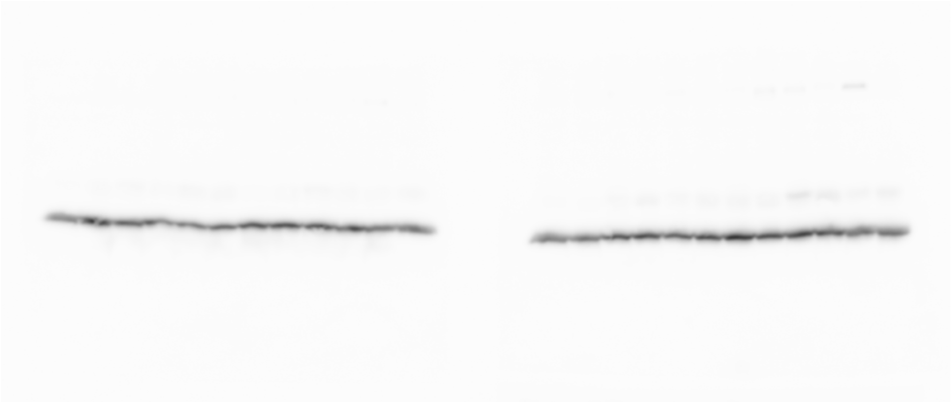

Dynamin 1

Supplementary Figure 6A

Total α-synuclein

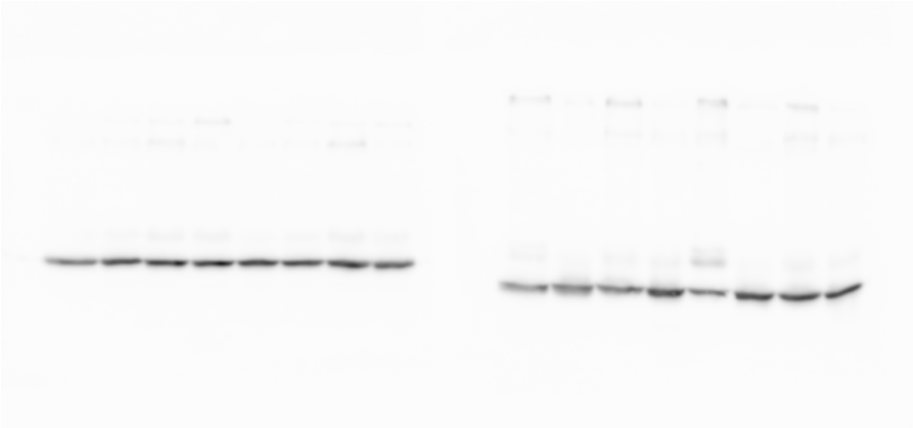

pRab10

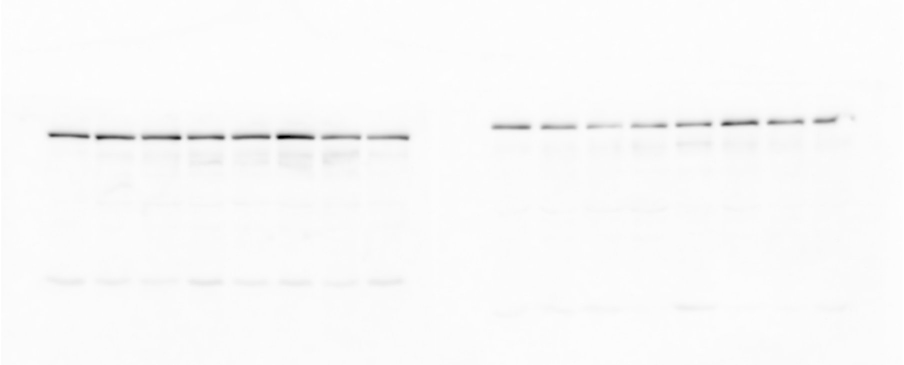

pRab12

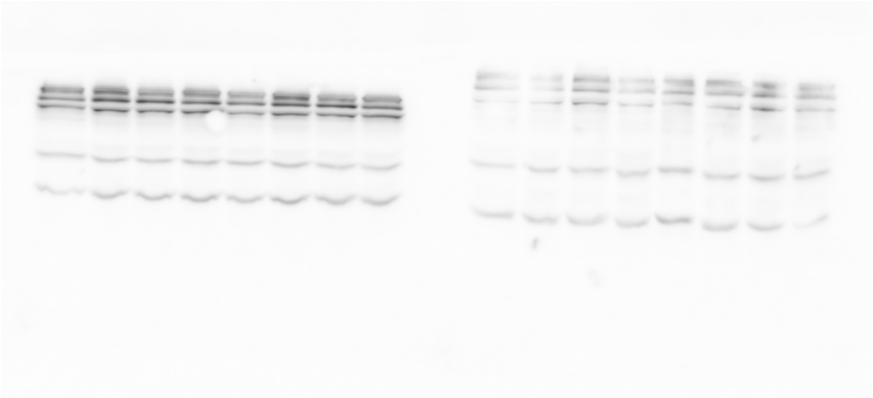

Total Rab10

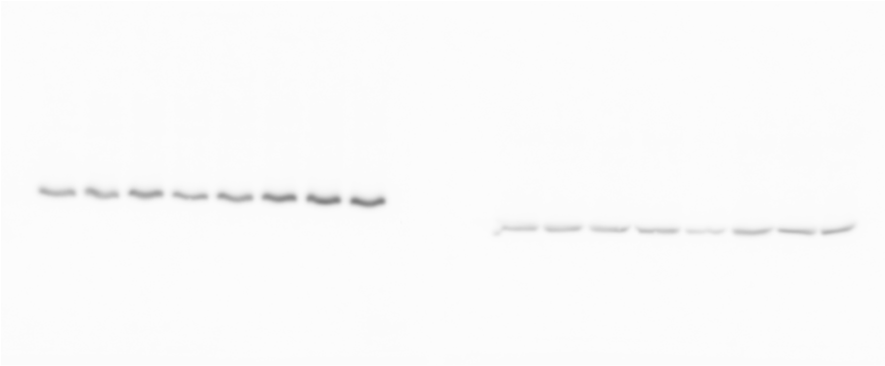

Total Rab12

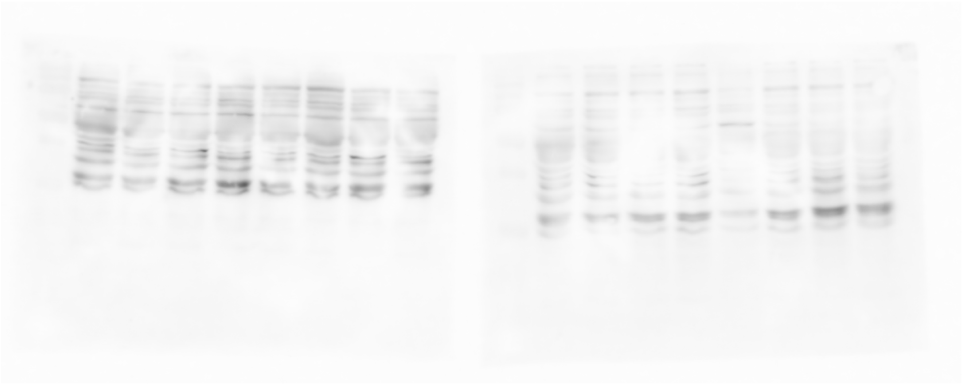

Ubiquitin

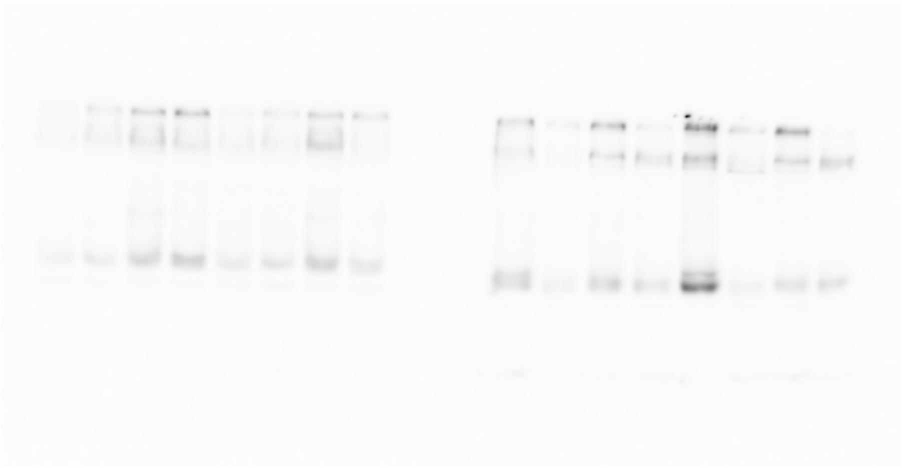

Actin

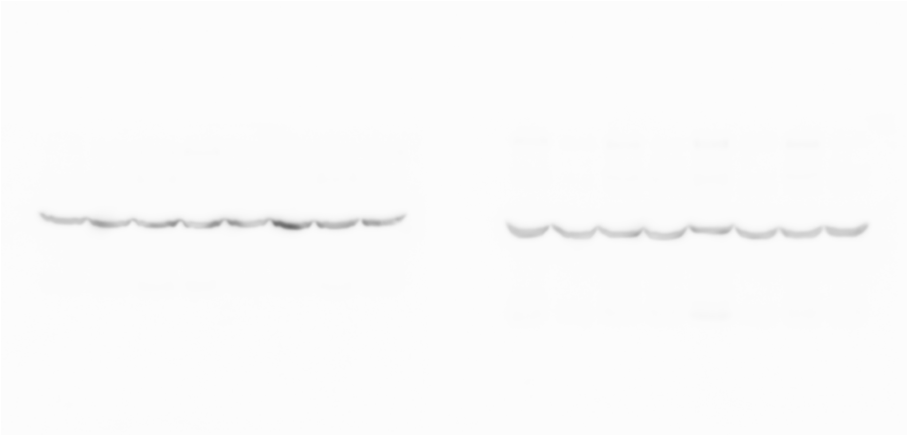

Supplementary Figure 6B

Cathepsin D

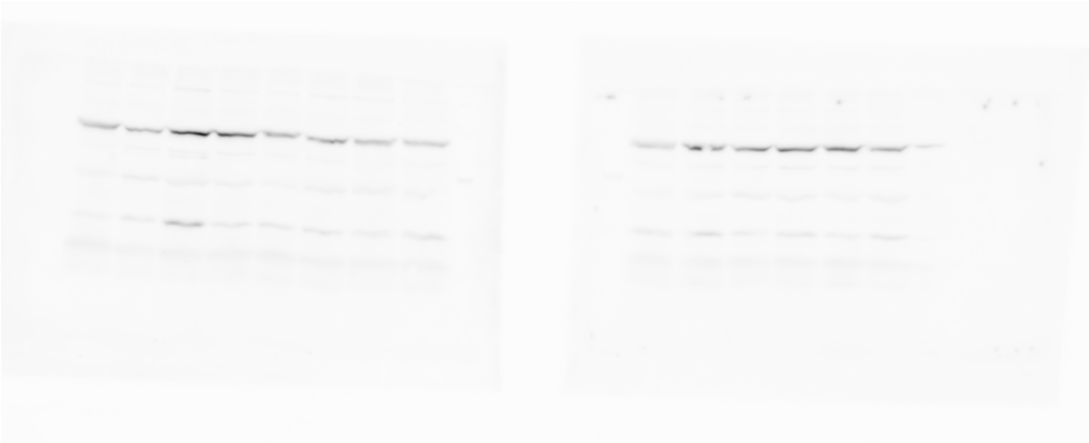

LAMP2

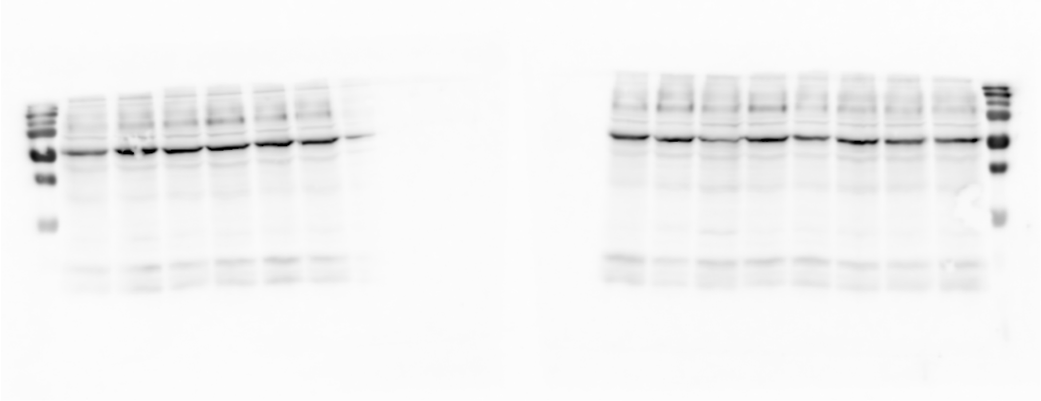

LC3B

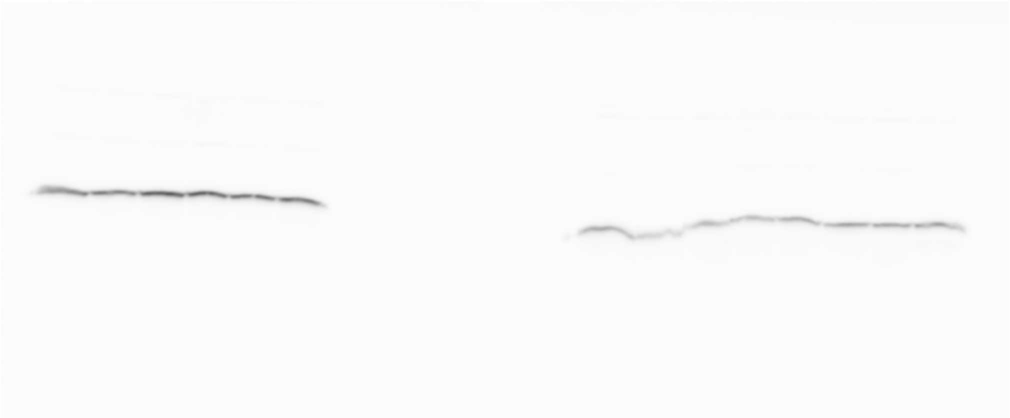

p62

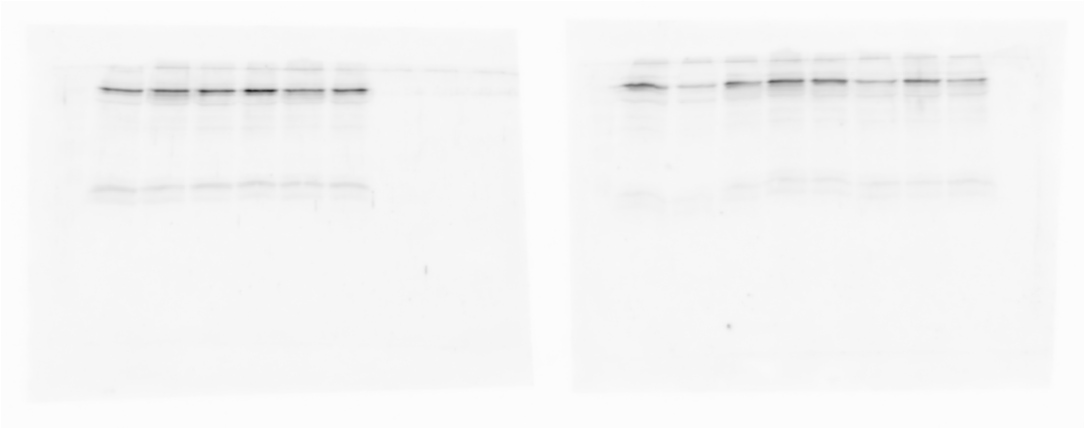

Dynamin 1

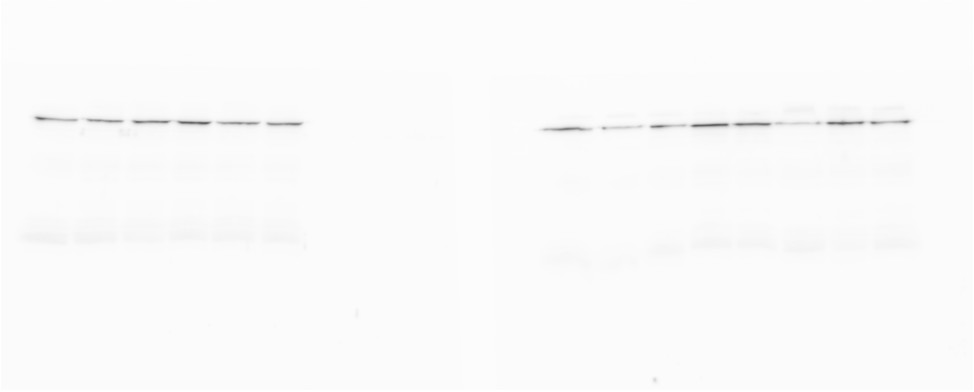

Supplement: Supplementary file 1 — Supplementary Material [file 41531_2024_748_MOESM1_ESM.pdf]
